# Asia‐enriched survey of spatial biology and three‐dimensional pathology

**DOI:** 10.1002/imt2.70187

**Published:** 2026-11-04

**Authors:** Zhengyang Wang, Zhu Zhu, Ruisi Li, Rachel Elizabeth Fincham, Andrew Song, Timothy Tay, Yuan Wang, Yang Wu, Denise Goh, Timothy Wang, Felicia Wee, Rajiv Kumar Kaushal, Ilknur Turkmen, Yosep Chong, Vincenzo L'Imperio, Sabine Leh, David Ameisen, Diana Montezuma, Jordi Temprana‐Salvador, Mircea‐Sebastian Serbanescu, Sebastian Marwitz, Etsuo Susaki, Nadieh Khalili, Anil Parwani, Norman Zerbe, Jasmine Plummer, Carlo Bifulco, Chee Leong Cheng, Shenglei Li, Inti Zlobec, Haeyoun Kang, Andrey Bychkov, Junya Fukuoka, Joe Yeong

**Affiliations:** ^1^ Institute of Molecular and Cell Biology (IMCB) Agency for Science, Technology and Research (A*STAR) Singapore Republic of Singapore; ^2^ Department of Pathology The First Affiliated Hospital of Zhengzhou University Zhengzhou China; ^3^ Department of Biological Sample Bank The First Affiliated Hospital of Zhengzhou University Zhengzhou China; ^4^ Department of Anatomical Pathology Singapore General Hospital Singapore Singapore; ^5^ Department of Pathology Brigham and Women's Hospital, Harvard Medical School Boston Massachusetts USA; ^6^ Department of Pathology Tata Memorial Hospital, Homi Bhabha National Institute (HBNI) Mumbai India; ^7^ Department of Pathology Memorial Healthcare Group Istanbul Turkey; ^8^ Department of Hospital Pathology The Catholic University of Korea Uijeongbu South Korea; ^9^ Department of Medicine and Surgery, Pathology, San Gerardo Hospital University of Milano‐Bicocca Monza Italy; ^10^ Department of Pathology Haukeland University Hospital Bergen Norway; ^11^ Imginit SAS, 152 Boulevard du Montparnasse Paris France; ^12^ European Society of Digital and Integrative Pathology (ESDIP) Lisbon Portugal; ^13^ Department of Pathology Vall d'Hebron University Hospital Barcelona Spain; ^14^ Department of Medical Informatics and Biostatistics University of Medicine and Pharmacy Craiova Romania; ^15^ Histology, Research Center Borstel‐Leibniz Lung Center Borstel Germany; ^16^ Airway Research Center North (ARCN), Member of the German Center for Lung Research Großhansdorf Germany; ^17^ Department of Biochemistry and Systems Biomedicine Juntendo University Tokyo Japan; ^18^ Computational Pathology Group, Department of Pathology Radboud University Medical Center Nijmegen The Netherlands; ^19^ Department of Pathology Ohio State University Columbus Ohio USA; ^20^ Institute of Pathology University Medical Center Hamburg‐Eppendorf Hamburg Germany; ^21^ Institute of Medical Informatics, Charité ‐ Universitätsmedizin Berlin Berlin Germany; ^22^ Center for Spatial Omics, St. Jude Children's Research Hospital Memphis Tennessee USA; ^23^ Providence Portland Medical Center Portland Oregon USA; ^24^ Institute of Tissue Medicine and Pathology University of Bern Bern Switzerland; ^25^ Department of Digital Medicine University of Bern Bern Switzerland; ^26^ Department of Pathology, CHA Bundang Medical Center CHA University Seongnam South Korea; ^27^ Department of Pathology Kameda Medical Center Kamogawa Japan; ^28^ Department of Pathology Informatics Nagasaki University Graduate School of Biomedical Sciences Nagasaki Japan; ^29^ The First Affiliated Hospital of Wenzhou Medical University Wenzhou China; ^30^ School of Basic Medical Science Wenzhou Medical University Wenzhou China; ^31^ Division of Cancer Signalling and Therapy, Institute of Molecular and Cell Biology (IMCB), Agency for Science, Technology and Research (A*STAR) Singapore Republic of Singapore; ^32^ Division of Pathology Singapore General Hospital Singapore Singapore; ^33^ Zhejiang Key Laboratory of Intelligent Cancer Biomarker Discovery and Translation The First Affiliated Hospital of Wenzhou Medical University Wenzhou China; ^34^ Duke‐NUS Medical School Singapore Singapore; ^35^ Department of Microbiology Singapore General Hospital Singapore Singapore

## Abstract

An Asia‐enriched survey of 239 respondents found that reported implementation of spatial biology and three‐dimensional pathology varied across self‐rated experience groups. Respondents identified cost, analytical complexity, limited standardization, and insufficient interdisciplinary expertise as barriers, highlighting the needs for practical workflows and computational support.

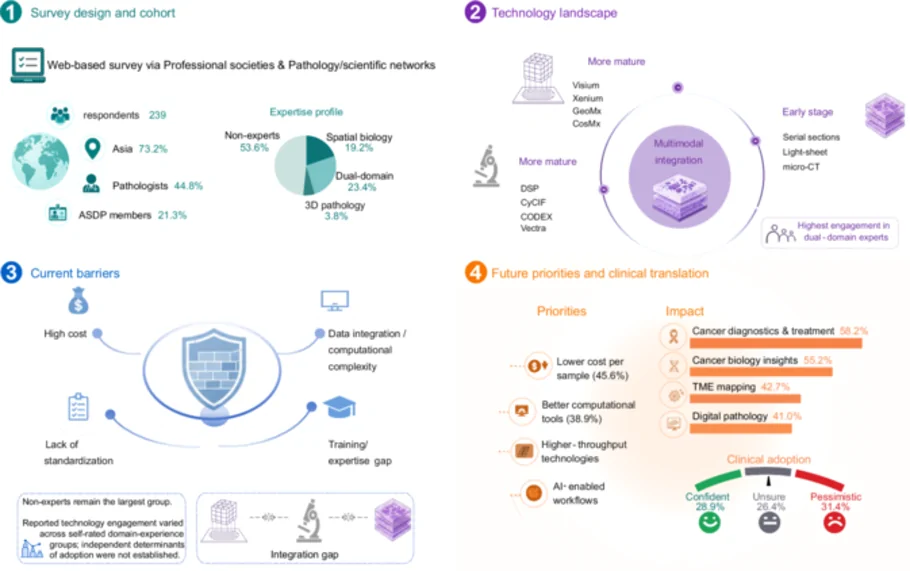

To the Editor,

Spatial biology and three‐dimensional (3D) pathology are transforming tissue‐based research by providing complementary views of tissues in their native spatial context [1, 2]. The underlying spatial transcriptomic and proteomic technologies enable high‐resolution mapping of molecular expression and cellular interactions within the tissue microenvironment, extending traditional molecular profiling beyond dissociated or bulk tissue analysis [1, 3]. In parallel, 3D pathology overcomes the limitations of thin two‐dimensional (2D) sections by enabling volumetric visualization of tissue architecture, stromal networks, invasion patterns, and diagnostically relevant structures that may be missed or misinterpreted using conventional histology [4, 5, 6].

These approaches inform “tissue intelligence,” integrating molecular profiling with spatial, morphological, and volumetric information [7, 8, 9]. Literature describes implementation challenges involving cost, workflow and analytical complexity, standardization, and specialized expertise [10, 11]. These reports contextualize respondents' perceived needs; this survey does not estimate implementation in the broader professional population.

We conducted a cross‐sectional, self‐administered online survey of 239 respondents in an Asia‐enriched, non‐probability convenience sample (Figure 1A). An international multidisciplinary panel of more than 10 pathologists and domain experts developed the questionnaire and interpreted findings. Several held current or previous roles in the Asian Society of Digital Pathology (ASDP), the European Society of Digital and Integrative Pathology (ESDIP), and related societies. Self‐rated spatial biology and 3D pathology experience (≥3 for higher experience in each domain) defined four operational groups: spatial biology experts, 3D pathology experts, dual‐domain experts, and non‐experts. These labels do not denote independently validated expertise; non‐experts had lower classified experience in both domains. Reporting followed the Consensus‐Based Checklist for Reporting of Survey Studies (CROSS) [12]. Detailed methods, grouping criteria, and the questionnaire are in the Supporting Information; Comparisons between early and late respondents are presented in Table S1.

**FIGURE 1:**
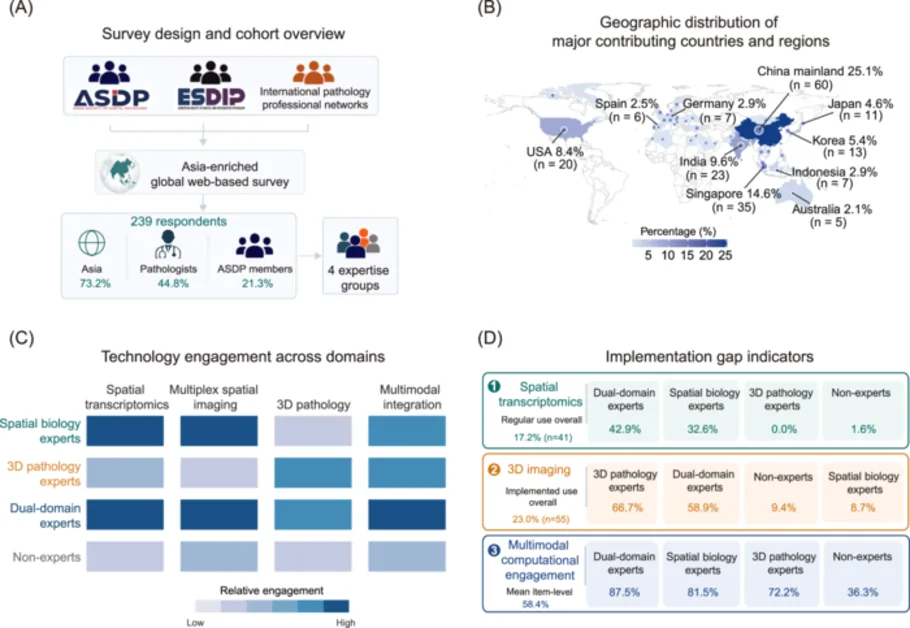
Survey cohort, geographic distribution, and expertise‐stratified adoption landscape. (A) Survey design and cohort overview of the Asia‐enriched global web‐based survey. The survey involved participation by members of the Asian Society of Digital Pathology (ASDP) and the European Society of Digital and Integrative Pathology (ESDIP). Society logos are used with permission to denote member participation. (B) Geographic distribution of the leading contributing countries/regions, with respondent counts and percentages. (C) Relative engagement in spatial transcriptomics, multiplex spatial imaging, three‐dimensional (3D) pathology, and multimodal integration, stratified by expertise group. (D) Implementation indicators for regular use of spatial transcriptomics, implemented use of 3D imaging, and the mean item‐level engagement indicator for Questions 6.1 and 6.2. The latter is calculated as 100 × (Question 6.1‐positive respondents + Question 6.2‐positive respondents)/(2 × group size), with respondents counted once per item. No feedback contributes no recorded positive and remains in the denominator; the indicator is not a percentage of unique engaged respondents.

## SURVEY COHORT AND EXPERTISE COMPOSITION

The survey included 239 respondents. Most were based in Asia (73.2%, *n* = 175), primarily mainland China (25.1%, *n* = 60), Singapore (14.6%, *n* = 35), and India (9.6%, *n* = 23) (Figure 1B; Figure S1A). Respondents were classified as spatial biology experts (19.2%), 3D pathology experts (3.8%), dual‐domain experts (23.4%), or non‐experts (53.6%) (Figure S1B). ASDP members accounted for 21.3% (*n* = 51) (Figure S1C). Pathologists represented the largest professional group (44.8%, *n* = 107) (Figure S1D).

Classification as dual‐domain experts was more frequent among responding industry professionals (42.9%) and data scientists (38.5%) than pathologists (19.6%) (Figure S1D). Among non‐experts, 41.4% had more than 10 years of professional experience, indicating that lower domain ratings did not imply limited career experience (Table S2). Overall career experience is shown in Figure S2A. Non‐experts predominated in hospital settings, whereas expert groups were more concentrated in industry (Figure S2B; Table S2). Grouping used self‐rated domain scores (Figure S2C–E).

## EXPERTISE‐STRATIFIED ADOPTION ACROSS TECHNOLOGY DOMAINS

Reported familiarity, implementation, and computational support varied across self‐rated experience groups (Figure 1C). Visium (*n* = 104), Xenium (*n* = 63), and GeoMx (*n* = 59) were the most frequently reported spatial molecular profiling platforms, particularly among spatial biology and dual‐domain experts (Figure S3A–B; Table S3). Multiplex platforms included digital spatial profiling, cyclic immunofluorescence (CyCIF), and co‐detection by indexing (CODEX)/PhenoCycler. Dual‐domain experts more frequently reported higher multiplexing capacity; non‐experts generally reported lower marker ranges or no relevant experience (Figure S4A–B; Table S4).

Serial section‐based reconstruction (32.6%), light‐sheet microscopy (21.8%), and 3D immunohistochemistry/multiplex imaging (16.3%) were the most frequently reported 3D approaches; dual‐domain experts reported greater use and non‐experts less familiarity (Figure S5A–B; Table S5). The most frequently selected perceived advantages were preserving spatial context and cellular relationships for spatial transcriptomics (55.6%), tumor‐microenvironment characterization for multiplex imaging (45.6%), and understanding tissue architecture for 3D pathology (53.1%) (Figures S3C, S4C, and S5C). Reported multimodal approaches included multi‐omics integration (38.9%), artificial intelligence (AI)‐based analysis (31.4%), and image‐based integration (30.1%), with greater reporting by expert groups (Table S6). Computational support mainly involved in‐house bioinformatics teams and collaborations; non‐experts more frequently reported not analyzing spatial/3D data (Figure S6A–B).

## IMPLEMENTATION STAGES AND TRANSLATION GAP

Recorded implementation indicators varied across technology domains and self‐rated experience groups (Figure 1D). For spatial transcriptomics, regular use was reported by 41 of 239 respondents (17.2%). Group‐specific proportions were higher among dual‐domain experts (42.9%) and spatial biology experts (32.6%) than among non‐experts (1.6%). For 3D imaging, regular or occasional use was reported by 55 of 239 respondents (23.0%), comprising 66.7% of 3D pathology experts, 58.9% of dual‐domain experts, 9.4% of non‐experts, and 8.7% of spatial biology experts (Tables S5 and S7). These percentages use the full cohort or group as the denominator and describe recorded use. No feedback was recorded for 83 respondents (34.7%) in Question 5.2, as shown in Figure S5B and reported separately in Table S7, and was not interpreted as confirmed non‐implementation. A mean item‐level engagement indicator derived from Questions 6.1 and 6.2 was highest among dual‐domain experts (87.5%), followed by spatial biology experts (81.5%), 3D pathology experts (72.2%), and non‐experts (36.3%). This two‐item average uses full‐group denominators and is not a percentage of unique engaged respondents. These unadjusted summaries describe variation in reported use and engagement across the operational categories of self‐rated domain experience.

## BARRIERS AND DOMAIN‐SPECIFIC BOTTLENECKS

Across technology domains, major barriers included cost, computational complexity, limited standardization, sample preparation, restricted access, and insufficient expertise or training (Figure 2A). For spatial transcriptomics, the most frequently reported barriers were technology or reagent costs (*n* = 140) and complex data analysis and integration (*n* = 88); key barriers for multiplex imaging included antibody validation, analytical complexity, and signal‐to‐noise issues, whereas those for 3D pathology included equipment costs, image‐processing demands, and sample preparation (Figures S3D, S4D, and S5D). Thus, cost and computational complexity emerged as shared obstacles alongside modality‐specific bottlenecks (Figure 2B). These findings highlight priorities for broader translation, including lower costs, improved computational tools, standardized workflows, robust wet‐lab protocols, accessible infrastructure, and interdisciplinary training.

**FIGURE 2:**
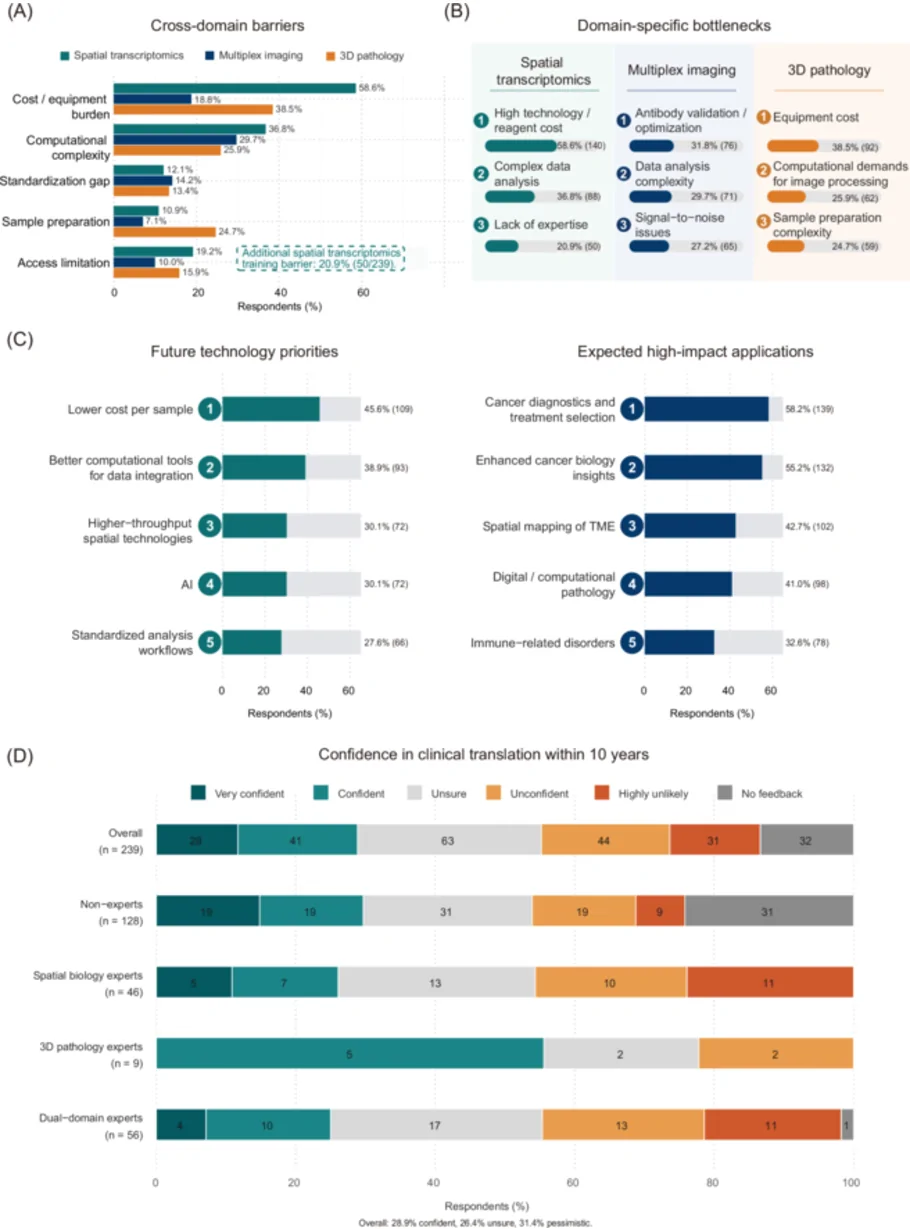
Barriers, bottlenecks, and translational priorities. (A) Cross‐domain barriers to the implementation of spatial transcriptomics, multiplex imaging, and three‐dimensional (3D) pathology. (B) Leading domain‐specific bottlenecks for spatial transcriptomics, multiplex imaging, and 3D pathology. (C) Future technological priorities and expected high‐impact biomedical applications. (D) Confidence in the clinical translation of spatial biology and 3D pathology within the next decade, stratified by expertise group. In panel D, no feedback is shown separately, and percentages use full‐cohort or full‐group denominators. Ordered confidence comparisons exclude no feedback and include 207 valid responses: 46 spatial biology experts, 9 3D pathology experts, 55 dual‐domain experts, and 97 non‐experts. AI, artificial intelligence; TME, tumor microenvironment.

## FUTURE PRIORITIES AND CLINICAL TRANSLATION

Respondents most frequently selected lower cost per sample (45.6%) and improved computational tools for integration (38.9%) in Question 6.3, followed by higher throughput and AI‐based approaches (Figure 2C; Figure S6C). Other selections included standardized workflows, clinical‐grade validation, improved resolution, and broader molecular coverage. Open‐ended responses mentioned user‐friendly interoperable tools, AI‐assisted integration of spatial omics, multiplex imaging, 3D pathology, hematoxylin and eosin (H&E)‐stained morphology, clinical data, and formalin‐fixed paraffin‐embedded (FFPE) compatibility. The most frequently selected expected applications were cancer diagnostics/treatment selection (58.2%), cancer biology (55.2%), tumor‐microenvironment mapping (42.7%), and digital/computational pathology (41.0%) (Figure 2C; Figure S6D). Regarding clinical adoption within the next decade, 28.9% were confident, 26.4% unsure, 31.4% pessimistic, and 13.4% provided no feedback (Figure 2D; Figure S6E; Table S6). Of the 32 respondents with no feedback, 31 were non‐experts (31/128, 24.2%) and one was a dual‐domain expert (1/56, 1.8%); the spatial biology and 3D pathology expert groups had complete feedback. These responses describe expectations, not forecasts.

## EXPLORATORY STATISTICAL ANALYSES

Early‐late respondents (120/119) differed in professional role and self‐rated experience after Benjamini–Hochberg (BH) and Benjamini–Yekutieli (BY) correction (Table S1). The non‐expert late‐minus‐early difference was 32.2 percentage points (pointwise 95% confidence interval (CI), 19.7–43.3). Experience composition also differed across four chronological blocks after correction. Among 84 exploratory comparisons (Tables S2–S6), 55 met BH and 42 BY thresholds; all are reported. Table S7 summarizes marker multiplexing and 3D imaging use. Comparisons without covariate adjustment cannot establish an independent contribution of experience to implementation. These chronological comparisons cannot quantify or exclude non‐response bias.

In summary, we surveyed an international, Asia‐enriched convenience sample of 239 respondents to characterize reported technology use and perceived implementation and translational challenges. Respondents reported familiarity with or use of multiple spatial biology platforms, with use varying across technology domains and self‐rated experience groups. The larger dual‐domain group (*n* = 56), compared with the 3D pathology–only group (*n* = 9), reflects co‐occurring higher self‐rated experience in both domains within this sample.

Reported barriers are consistent with literature on analytical and implementation challenges [10, 11]. However, experience and technology‐use questions are conceptually adjacent, and group patterns may also reflect professional role, work setting, region, career duration, society membership, and recruitment route. The survey cannot determine whether experience precedes implementation or develops through use. Computational methods summarized in Tables S8–S9 provide technical context for respondents' perceived needs. Spatial registration, which can be affected by section damage, deformation, and differences between modalities, should be distinguished from multimodal integration. The survey did not evaluate these computational methods or their effects on implementation.

Respondents valued multimodal integration but reported it unevenly, prioritizing improved computational tools. These needs align with broader computational challenges, including cell segmentation as a major bottleneck (Table S8). Virtual Alignment of pathoLogy Image Series and elastix primarily align morphological features; Probabilistic Alignment of Spatial Transcriptomics Experiments (PASTE)/PASTE2, STalign, Gaussian process spatial alignment, and Spatially‐Linked Alignment Tool (SLAT) also incorporate molecular, spatial, or graph‐based information. Robust multimodal 2D‐to‐3D alignment across pixel, cell, and voxel scales remains challenging (Table S9). Open‐ended responses identified translational needs for interdisciplinary expertise, scalable protocols, interoperable infrastructure, AI‐enabled interpretation, standardized validation, and demonstrated clinical utility.

In Xenium and CosMx, densely packed tissues with irregular or overlapping cells can complicate boundary detection, leading to transcript misassignment and impaired interpretation of cell–cell interactions [13]. In 3D pathology, depth‐dependent signal loss, tissue deformation, cell overlap, and registration errors can similarly disrupt segmentation consistency across image stacks and distort reconstruction. Available approaches include the morphology‐based methods Cellpose and Mesmer, transcript‐driven methods such as Baysor and ComSeg, multimodal methods including biologically informed deep learning‐based cell segmentation (BIDCell), RNA2seg, and diffusion‐based cell segmentation integrating spatially resolved transcriptome (DISSECT), the probabilistic method Proseg, and the volumetric method u‐Segment3D (Table S8). However, robust, reproducible, and clinically validated solutions remain limited.

FFPE compatibility remains central to clinical translation because FFPE specimens predominate in routine pathology and archival tissue collections. Fixation‐induced ribonucleic acid (RNA) fragmentation, cross‐linking, and variable molecular preservation can impair transcript detection and downstream analytical robustness [14]. However, FFPE is no longer a uniform limitation: Xenium and CosMx support targeted in situ workflows, whereas Visium CytAssist enables probe‐based profiling of FFPE and other fixed tissues. Compatibility is therefore increasingly platform‐ and assay‐specific, although background, transcript assignment, segmentation, and tissue variability remain important considerations.

Reported familiarity with or use of 3D imaging approaches varied by modality, led by serial section‐based reconstruction (32.6%) and light‐sheet microscopy (21.8%). Light‐sheet microscopy provides high‐resolution volumetric and molecular imaging but requires clearing, specialized instrumentation, and intensive processing. Optical coherence tomography (OCT) offers rapid, label‐free structural imaging with potential intraoperative applications; micro‐OCT approaches cellular‐scale resolution at the expense of volume and acquisition efficiency, whereas micro‐computed tomography (micro‐CT) enables nondestructive whole‐specimen X‐ray imaging [4, 15] (Table S10). These differences highlight modality‐ and use‐case‐specific validation needs; their contribution to reported implementation was not tested.

Interpretation is limited by unequal professional composition, possible bias in self‐rated experience classification, and the small 3D pathology expert subgroup (*n* = 9). Post hoc public‐information review of 15 selected, confidently matched respondents was descriptive and did not independently validate experience classifications in the full cohort. Open‐network recruitment prevented conventional response‐rate estimation, while early–late differences in professional role and self‐rated experience suggest possible non‐response bias (Table S1). Asia enrichment (73.2%) further limits generalizability. Only four respondents worked in government agencies, and regulators were not specifically targeted; future surveys should include more regulatory and governance stakeholders. For Question 6.5, no feedback was concentrated among non‐experts; confidence comparisons, therefore, describe respondents who answered the item and may be affected by selective response.

Translation of AI‐based analysis and multimodal integration requires attention to dataset representativeness. Pathology foundation models often rely on institutionally concentrated cohorts with limited international validation. Larger or multicenter datasets dominated by well‐resourced populations may improve average performance while masking disparities; Western validation may not ensure comparable performance in Asian or other underrepresented populations. Small cohorts in spatial studies may compound these concerns (Table S11). Future datasets should combine scale with diversity in geography, ancestry, disease, clinical settings, laboratories, scanners, tissue processing, and assay platforms, supported by subgroup evaluation and external validation. Spatial multi‐omics and 3D “digital twin” data also raise privacy, security, interoperability, and sharing concerns. Governance options include controlled access, encryption, auditability, federated analysis, differential privacy, consent‐linked restrictions, and rules for secondary use (Table S12) [16]. These considerations complement respondents' priorities for interdisciplinary training, standardized workflows, and improved computational support; their effects on clinical translation were not evaluated in this survey.

## AUTHOR CONTRIBUTIONS

**Zhengyang Wang**: Methodology; investigation; formal analysis; writing—original draft. **Zhu Zhu**: methodology; investigation; writing—original draft; formal analysis. **Ruisi Li**: Methodology; Investigation; formal analysis. **Rachel Elizabeth Fincham**: Methodology; Investigation; formal analysis. **Andrew Song**: Investigation; writing—review and editing. **Timothy Tay**: Investigation; writing—review and editing. **Yuan Wang**: Investigation; writing—review and editing. **Yang Wu**: Investigation; writing—review and editing. **Denise Goh**: Investigation; writing—review and editing. **Timothy Wang**: Investigation; writing—review and editing. **Felicia Wee**: Investigation; writing—review and editing. **Rajiv Kumar Kaushal**: Writing—review and editing. **Ilknur Turkmen**: Writing—review and editing. **Yosep Chong**: Writing—review and editing. **Vincenzo L'Imperio**: Writing—review and editing. **Sabine Leh**: Writing—review and editing. **David Ameisen**: Writing—review and editing. **Diana Montezuma**: Writing—review and editing. **Jordi Temprana‐Salvador**: Writing—review and editing. **Mircea‐Sebastian Serbanescu**: Writing—review and editing. **Sebastian Marwitz**: Writing—review and editing. **Etsuo Susaki**: Methodology. **Nadieh Khalili**: Methodology. **Anil Parwani**: Methodology. **Norman Zerbe**: Methodology. **Jasmine Plummer**: Methodology. **Carlo Bifulco**: Methodology. **Chee Leong Cheng**: Methodology; supervision. **Shenglei Li**: Methodology; supervision. **Inti Zlobec**: Methodology; supervision. **Haeyoun Kang**: Methodology; supervision; writing—review and editing. **Andrey Bychkov**: Methodology; supervision; writing—review and editing. **Junya Fukuoka**: Methodology; supervision; writing—review and editing. **Joe Yeong**: Methodology; supervision; writing—review and editing.

## CONFLICT OF INTEREST STATEMENT

The authors declare no conflicts of interest.

## ETHICS STATEMENT

Participation was voluntary, and questionnaire completion after disclosure of the study purpose constituted implied consent. Optional email addresses, names, and affiliations were excluded from the primary analyses and used only for limited post hoc matching to public professional records. The primary analyses used de‐identified data, and only aggregate findings are reported. Under local institutional policy, formal ethics approval was not required for this minimal‐risk survey.

## Supporting information


**Figure S1**: Regional distribution, expertise composition, Asian Society of Digital Pathology (ASDP) membership, and professional roles.
**Figure S2**: Respondent experience, work setting, and expertise classification.
**Figure S3**: Spatial molecular profiling technologies and spatial transcriptomics.
**Figure S4**: Multiplex spatial imaging and multiplex immunofluorescence landscape.
**Figure S5**: Three‐dimensional (3D) pathology modality landscape and implementation barriers.
**Figure S6**: Multimodal integration, computational support, future priorities, and clinical translation confidence.

**Table S1**: Early‐late respondent comparison and sensitivity to chronological grouping.
**Table S2**: Comparisons of respondent characteristics across expertise categories.
**Table S3**: Spatial molecular profiling technologies and spatial transcriptomics: group comparisons across expertise categories.
**Table S4**: Multiplex immunofluorescence (mIF): group comparisons across expertise categories.
**Table S5**: Three‐dimensional (3D) pathology: group comparisons across expertise categories.
**Table S6**: Multimodal integration and clinical translation confidence: group comparisons across expertise categories.
**Table S7**: Descriptive summaries of marker multiplexing and reported three‐dimensional imaging use by self‐rated experience group.
**Table S8**: Representative computational and artificial intelligence (AI)‐based cell‐segmentation approaches relevant to spatial transcriptomics and three‐dimensional (3D) pathology.
**Table S9**: Representative computational tools for registration and alignment in spatial multi‐omics and three‐dimensional (3D) pathology.
**Table S10**: Comparison of major volumetric imaging approaches relevant to three‐dimensional (3D) pathology.
**Table S11**: Representative recent high‐impact studies illustrating dataset scale and geographic representation in artificial intelligence (AI) pathology and spatial biology.
**Table S12**: Potential strategies for privacy‐preserving governance of spatial multi‐omics and three‐dimensional (3D) pathology data.

## Data Availability

The summary statistics supporting the findings of this study are provided in the article and its Supporting Information. De‐identified respondent‐level survey data are publicly available on GitHub at https://github.com/zhuzhu1986/Wang-Zhengyang. The full survey questionnaire is included in the Supporting Information. Names, email addresses, affiliations, and other identifying information are excluded from the shared data to protect participant confidentiality. Supplementary materials (figures, tables, methods, graphical abstract, slides, videos, Chinese translated version, and updated materials) may be found in the online DOI or iMeta Science http://www.imeta.science/.
