# Supplementary material for: Asia‐enriched survey of spatial biology and three‐dimensional pathology
Source: Imeta. 2026 Nov 4:e70187. Online ahead of print. doi: 10.1002/imt2.70187 (PMC13733675; doi:10.1002/imt2.70187)
Supplement: Supplementary file 1 [file IMT2-9999-e70187-s001.docx]

Supporting Information to

**Asia-enriched survey of spatial biology and three-dimensional pathology**

**Running title**: Spatial biology and three-dimensional pathology survey

Zhengyang Wang^1,2#^, Zhu Zhu^3#^, Ruisi Li^1#^, Rachel Elizabeth Fincham^4#^, Andrew Song^5^, Timothy Tay^4^, Yuan Wang^2^, Yang Wu^1^, Denise Goh^1^, Timothy Wang^1^, Felicia Wee^1^, Rajiv Kumar Kaushal^6^, Ilknur Turkmen^7^, Yosep Chong^8^, Vincenzo L'Imperio^9^, Sabine Leh^10^, David Ameisen^11^, Diana Montezuma^12^, Jordi Temprana-Salvador^13^, Mircea-Sebastian Serbanescu^14^, Sebastian Marwitz^15,16^, Etsuo Susaki^17^, Nadieh Khalili^18^, Anil Parwani^19^, Norman Zerbe^20,21^, Jasmine Plummer^22^, Carlo Bifulco^23^, Chee Leong Cheng^4^, Shenglei Li^2^, Inti Zlobec^24,25^, Haeyoun Kang^26*^, Andrey Bychkov^27*^, Junya Fukuoka^28*^, Joe Yeong^29,30,31,32,33,34,35*^

^1^Institute of Molecular and Cell Biology (IMCB), Agency for Science, Technology and Research (A*STAR), Singapore 138673, Republic of Singapore

^2^Department of Pathology, The First Affiliated Hospital of Zhengzhou University, Zhengzhou 450052, China

^3^Department of Biological Sample Bank, The First Affiliated Hospital of Zhengzhou University, Zhengzhou 450052, China

^4^Department of Anatomical Pathology, Singapore General Hospital, Singapore 169856, Singapore

^5^Department of Pathology, Brigham and Women's Hospital, Harvard Medical School, Boston, MA 02115, United States

^6^Department of Pathology, Tata Memorial Hospital, Homi Bhabha National Institute (HBNI), Mumbai 400012, India

^7^Department of Pathology, Memorial Healthcare Group, Istanbul 34385, Turkey

^8^Department of Hospital Pathology, The Catholic University of Korea, Uijeongbu 11765, South Korea

^9^Department of Medicine and Surgery, Pathology, San Gerardo Hospital, University of Milano-Bicocca, Monza 20900, Italy

^10^Department of Pathology, Haukeland University Hospital, Bergen 5021, Norway

^11^Imginit SAS, 152 Boulevard du Montparnasse, Paris 75014, France

^12^European Society of Digital and Integrative Pathology (ESDIP), Lisbon 1050‑012, Portugal

^13^Department of Pathology, Vall d'Hebron University Hospital, Barcelona 08035, Spain

^14^Department of Medical Informatics and Biostatistics, University of Medicine and Pharmacy, Craiova 200349, Romania

^15^Histology, Research Center Borstel-Leibniz Lung Center, Borstel 23845, Germany

^16^Airway Research Center North (ARCN), Member of the German Center for Lung Research, Großhansdorf 22927, Germany

^17^Department of Biochemistry and Systems Biomedicine, Juntendo University, Tokyo 113‑8421, Japan

^18^Computational Pathology Group, Department of Pathology, Radboud University Medical Center, Nijmegen 6525 GA, The Netherlands

^19^Department of Pathology, Ohio State University, Columbus, OH 43210, United States

^20^Institute of Pathology, University Medical Center Hamburg-Eppendorf, Hamburg 20246, Germany

^21^Institute of Medical Informatics, Charité - Universitätsmedizin Berlin, Berlin 10117, Germany

^22^Center for Spatial Omics, St. Jude Children's Research Hospital, Memphis, TN 38105‑3678, United States

^23^Providence Portland Medical Center, Portland, OR 97213, United States

^24^Institute of Tissue Medicine and Pathology, University of Bern, Bern 3008, Switzerland

^25^Department of Digital Medicine, University of Bern, Bern 3008, Switzerland

^26^Department of Pathology, CHA Bundang Medical Center, CHA University, Seongnam 13496, South Korea

^27^Department of Pathology, Kameda Medical Center, Kamogawa 296‑8602, Japan

^28^Department of Pathology Informatics, Nagasaki University Graduate School of Biomedical Sciences, Nagasaki 852‑8523, Japan

^29^The First Affiliated Hospital of Wenzhou Medical University, Wenzhou 325015, China

^30^School of Basic Medical Science, Wenzhou Medical University, Wenzhou 325035, China

^31^Division of Cancer Signalling and Therapy, Institute of Molecular and Cell Biology (IMCB), Agency for Science, Technology and Research (A*STAR), Singapore 138673, Republic of Singapore.

^32^Division of Pathology, Singapore General Hospital, Singapore 169856, Singapore

^33^Zhejiang Key Laboratory of Intelligent Cancer Biomarker Discovery and Translation, The First Affiliated Hospital of Wenzhou Medical University, Wenzhou 325015, China

^34^Duke-NUS Medical School, Singapore 169857, Singapore

^35^Department of Microbiology, Singapore General Hospital, Singapore 169856, Singapore

^#^These authors contributed equally: Zhengyang Wang, Zhu Zhu, Ruisi Li, and Rachel Elizabeth Fincham.

^*^Correspondence: yeongpohsheng@gmail.com (Joe Yeong); junfkoka@gmail.com (Junya Fukuoka); bychkov.andrey@kameda.jp (Andrey Bychkov); [hykang@cha.ac.kr](mailto:hykang@cha.ac.kr) (Haeyoun Kang).

## **METHODS**

**Study design and reporting**

This cross-sectional, web-based survey was conducted to evaluate the current status, adoption patterns, challenges, and future directions of spatial biology and three-dimensional (3D) pathology. The study was designed and reported in accordance with the Consensus-Based Checklist for Reporting of Survey Studies (CROSS) guidelines [1].

The survey initiative was led by members of the Asian Society of Digital Pathology (ASDP), with participation from members of the European Society of Digital and Integrative Pathology (ESDIP) and other international pathology communities. The target population included pathologists, pathology residents, clinicians, researchers, biomedical engineers, data scientists, bioinformaticians, industry professionals, medical students, trainees, and other stakeholders involved in or interested in spatial biology, digital pathology, 3D pathology, and related fields.

**Questionnaire development**

The questionnaire was developed through an iterative, expert-driven process informed by domain knowledge and relevant literature. It comprised seven sections: respondent characteristics; awareness and experience; spatial molecular profiling technologies, spatial transcriptomics implementation, advantages, and challenges; multiplex spatial imaging use and limitations; 3D pathology technologies, applications, and barriers; multimodal integration, computational analysis, and future perspectives; and optional additional comments. The questionnaire included single-choice, multiple-choice, Likert-scale, and open-ended questions. Conditional branching allowed respondents without relevant experience to skip selected technology-specific sections. The full survey questionnaire is reproduced below in the Supporting Information. Question 3.1 assessed familiarity with or use of a mixed technology list that included spatial assay for transposase-accessible chromatin using sequencing (ATAC-seq), which measures chromatin accessibility rather than transcript expression. The original questionnaire is reproduced without retrospective changes to its wording.

Respondents were grouped according to their self-rated experience in spatial biology (Question 2.2) and 3D pathology (Question 2.3), using a score of ≥ 3 as an operational cutoff for higher self-rated experience in each domain. The numeric midpoint (3) of the 1-5 scale provided an operational boundary for comparing domain-experience combinations. The four groups were termed “spatial biology experts” (Question 2.2 ≥ 3 and Question 2.3 < 3), “3D pathology experts” (Question 2.2 < 3 and Question 2.3 ≥ 3), “dual-domain experts” (both scores ≥ 3), and “non-experts” (both scores < 3, indicating lower self-rated experience in both surveyed domains). These terms are operational survey labels and do not indicate independently validated expertise. The groups were used for descriptive comparisons of reported technology familiarity, use, and implementation. Following the post hoc review, relevant-domain coauthors Junya Fukuoka, Joe Yeong, Andrey Bychkov, Vincenzo L'Imperio, Inti Zlobec, Norman Zerbe, Jordi Temprana-Salvador, Etsuo Susaki, Jasmine Plummer, and Carlo Bifulco discussed the findings and confirmed retention of the original grouping rule.

**Post hoc assessment of self-reported expertise**

A post hoc plausibility assessment was conducted among respondents who completed both Questions 2.2 and 2.3 and could be matched with high confidence to a unique professional identity. Respondents could choose to provide an email address, which was used only as an initial matching cue and corroborated using publicly available information, including names, institutional affiliations, departments, countries of practice, official institutional profiles, ORCID records, and publication affiliations. Identity was not inferred from an email address alone.

For high-confidence matches, publicly available information on professional roles, research fields, career histories, institutional profiles, ORCID records, and publications was reviewed and compared descriptively with the Questions 2.2 and 2.3 ratings. Cases were classified as concordant when documented professional activity supported the reported domain and relative pattern of self-rated experience, indeterminate when the available information was insufficiently field-specific, and discordant when it clearly conflicted with the self-ratings. Public professional information was concordant with the self-rating pattern for 13 of 15 respondents (86.7%); two cases were indeterminate, and none was discordant. This selected review was a descriptive plausibility assessment and did not independently validate expertise classifications in the full cohort.

Where an ORCID-linked OpenAlex author profile could be verified against the respondent’s name and institutional or publication information, the h-index and number of indexed works were recorded as ancillary descriptive indicators (OpenAlex; accessed August 23, 2026). Ambiguous profiles were treated as unavailable, and missing values were not imputed. This post hoc assessment was not used to define or reclassify expertise and did not alter the primary analyses. Personal identifiers were stored separately from the de-identified analytical dataset and used solely for identity matching; no names, email addresses, or respondent-level professional information were disclosed.

**Survey distribution and administration**

The survey was distributed through multiple channels, including professional societies, academic mailing lists, and direct invitations through international professional networks, including ASDP and ESDIP. Participation was voluntary, and no financial incentives were provided.

Provision of an email address was optional, and respondents could complete and submit the survey without providing one.

The survey was administered using two online platforms to improve accessibility across regions: Google Forms for respondents outside mainland China and Qualtrics for those in mainland China. Both versions contained identical questions, response options, branching logic, and survey instructions. Responses from the two platforms were exported, checked for consistency, merged, and analyzed as a single dataset. Each participant was limited to a single submission to minimize duplicate responses. The survey remained open from July 2025 to April 2026, and responses were automatically recorded and exported for analysis.

A total of 239 responses were received and included in the final analysis. Because the survey was distributed through open professional networks, professional societies, and mailing lists rather than through a closed sampling frame, the exact number of individuals who received the survey invitation could not be determined. Consequently, the denominator required to calculate a formal response rate was unavailable.

Country/region responses were standardized before analysis and grouped into predefined geographic regions. Türkiye and Israel were classified as Asia, whereas two responses that could not be reliably assigned to a country or region were categorized as unknown.

**Exploratory assessment of response timing and potential non-response bias**

We used an early-versus-late respondent comparison as an exploratory assessment of response timing, informed by the continuum-of-resistance model [2]. Recorded submission timestamps from the two survey platforms were converted to a common time zone, and the pooled responses were sorted by their actual submission times. The first 120 of the 239 respondents were classified as early respondents and the remaining 119 as late respondents, yielding approximately equal-sized chronological groups. The split was based on calendar submission time rather than predefined reminder waves or elapsed time since an individual invitation. Late respondents were not assumed to represent non-respondents. The comparison covered six characteristics: primary role, years of professional experience, primary work setting, geographic region, ASDP membership, and the operational self-rated experience groups defined using Questions 2.2 and 2.3. Experience classifications matched Table S2 (46/9/56/128 respondents). For sensitivity analysis, responses were also divided into four chronological blocks (60/60/60/59) and their experience-group distributions compared. Incomplete channel records precluded channel stratification.

For each of the 25 categories within these six characteristics, effect size was reported as the difference in proportions between late and early respondents, expressed in percentage points. Positive values indicate a higher proportion among late respondents. Pointwise 95% confidence intervals (CIs) were calculated using Newcombe’s method combining Wilson score intervals without continuity correction [3]. These descriptive intervals were not adjusted for multiplicity, do not account for selection or non-response, and were not used to declare category-level statistical significance. The six early-late tests and four-block experience comparison formed one seven-test family; no category-level hypothesis tests were performed. Uncorrected P values and Benjamini-Hochberg and Benjamini-Yekutieli adjusted P values are reported for all seven tests, with the correction methods specified under Statistical analysis. The complete counts, proportions, differences, CIs, and test results are provided in Table S1.

Professional role and self-rated experience in the early–late analysis, and experience composition in the four-block analysis, met both multiplicity thresholds, whereas the other four overall comparisons did not. Failure to meet a threshold was not interpreted as evidence of equivalence or absence of non-response bias. Because direct information on non-respondents was unavailable, the comparison could describe changes among respondents but could not determine the magnitude or direction of non-response bias. All cohort summaries and descriptive comparisons across self-rated experience groups were therefore interpreted as potentially affected by non-response bias.

**Statistical analysis**

The core analyses were descriptive comparisons across operational categories of self-rated domain experience. Survey responses were summarized as frequencies and percentages, generally using the full cohort or group as the denominator unless otherwise specified. Optional free-text responses to Question 7.1 were reviewed descriptively and grouped into recurring themes. Because these responses were exploratory and were not collected through a formal qualitative interview design, they were summarized narratively rather than subjected to formal qualitative coding. Table S7 presents descriptive summaries of marker multiplexing (Question 4.2) and recorded regular or occasional 3D imaging use (Question 5.2). For Question 4.2, marker-count categories, “not applicable to my work,” and “no feedback” were reported separately, using counts in Table S4. Marker-category proportions were also summarized among respondents with recorded feedback, including “not applicable” responses. For Question 5.2, reported regular or occasional use, other recorded responses, and no feedback were summarized separately using the aggregate counts reported in Table S5 and Figure S5B. Reported-use proportions were presented both for the full cohort or group and for respondents with recorded feedback. No feedback was not interpreted as confirmed non-implementation. Survey skips and unanswered items cannot be distinguished within these no-feedback summaries. These comparisons describe reported response patterns and do not establish independent or directional associations between self-rated experience and implementation.

The mean item-level engagement indicator in Figure 1D averages recorded engagement in Questions 6.1 (multimodal integration) and 6.2 (computational support). Respondents selecting at least one positive approach are counted once per item. Positive counts are obtained as group size minus “Not currently using multimodal integration” (Question 6.1) or “Not currently analyzing spatial/3D data” (Question 6.2), minus no feedback. The indicator (%) = 100 × (Question 6.1-positive respondents + Question 6.2-positive respondents)/(2 × group size). No feedback contributes no recorded positive and remains in the full-group denominator; this does not confirm non-use. Calculations use unrounded counts, with only final display values rounded to one decimal place. Question 6.1/6.2 positive counts are 35/40, 6/7, 49/49, and 50/43 for spatial biology experts, 3D pathology experts, dual-domain experts, and non-experts, respectively, yielding 81.5%, 72.2%, 87.5%, and 36.3% (overall: 279/478 = 58.4%). The indicator is not the proportion of unique respondents engaged in either or both activities.

For the multiplicity reanalysis, P values were recalculated from the tabulated group counts before correction. Pearson chi-square tests were used when all expected cell counts were ≥ 1 and at least 80% were ≥ 5. Otherwise, Fisher’s exact test was used for 2×2 tables and the Fisher-Freeman-Halton test with Monte Carlo sampling for larger tables. Each multiple-select option was compared as a recorded selection versus the remaining respondents within each group. The remainder may include skipped or unanswered items, so these tests concern recorded response patterns. Ordered category counts for years of experience (Question 1.2), marker multiplexing (Question 4.2), and clinical translation confidence (Question 6.5) were compared using tie-corrected Kruskal–Wallis statistics with Monte Carlo permutation calibration. “Not applicable” and “no feedback” were excluded from ordered-score tests. Question 4.2 had 122 valid marker-count responses (group counts: 38, 2, 43, and 39), and Question 6.5 had 207 valid responses (46, 9, 55, and 97), in the group order used in the tables. Monte Carlo tests used 1,000,000 samples. For Tables S2-S6, seeds were 20261006 + 1000 × table number + worksheet row. The S1 experience comparisons used fixed-margin Fisher–Freeman–Halton tests, ordered by table probability, with seeds 20261207 (early–late) and 20261407 (four-block). Monte Carlo P values were calculated as (b + 1)/(1,000,001), where b is the number of sampled statistics at least as extreme as observed.

All 84 exploratory comparisons in Tables S2-S6 (77 option-level and seven overall comparisons) were treated as one family and subjected to Benjamini-Hochberg (BH) correction of the false discovery rate (FDR) at 0.05 [4]. The six early–late comparisons and one four-block comparison in Table S1 formed a separate seven-test family addressing respondent composition over time. Benjamini-Yekutieli (BY) correction was also applied within each family as a more conservative sensitivity analysis for dependence among tests [5]. Correction used the recalculated P values at full available precision, not rounded display values. Uncorrected and corrected P values are reported for every comparison. Uncorrected P < 0.05 was treated only as a nominal exploratory result. BH-adjusted P < 0.05 was the primary multiplicity threshold; BY-adjusted P < 0.05 identified findings retained under the sensitivity analysis. Counts, proportions, and subject relevance guided reporting rather than the smallest P values. Multiplicity control was added during revision and does not constitute adjustment for professional or recruitment characteristics. All comparisons remain exploratory.

The original analyses and figures used SPSS 21.0, R (version 4.4.0), X-Tile (version 3.6.1), and GraphPad Prism 8. The aggregate-count reanalysis and multiplicity corrections were performed using Python 3.12.14 and SciPy 1.17.0.

**SUPPLEMENTARY FIGURES**

**Figure S1. Regional distribution, expertise composition, ASDP membership, and professional roles.** (A) Regional distribution of respondents based on country or region of practice. (B) Composition of self-reported expertise groups: spatial biology experts, 3D pathology experts, dual-domain experts, and non-experts. (C) ASDP membership status overall and by expertise group. (D) Distribution of primary professional roles overall and by expertise group.

**Figure S2. Respondent experience, work setting, and expertise classification.** (A) Years of professional experience. (B) Primary work setting. (C) Self-rated spatial biology expertise. (D) Self-rated 3D pathology expertise. (E) Distribution of predefined expertise groups: spatial biology experts, 3D pathology experts, dual-domain experts, and non-experts.

**Figure S3. Spatial molecular profiling technologies and spatial transcriptomics.** (A) Familiarity with or use of listed technologies (Question 3.1), including spatial assay for transposase-accessible chromatin using sequencing (ATAC-seq), which measures chromatin accessibility. (B) Spatial transcriptomics implementation by expertise group. (C) Perceived advantages. (D) Implementation challenges. Abbreviations: Spatial Molecular Imager (SMI); multiplexed error-robust fluorescence in situ hybridization (MERFISH); spatial enhanced resolution omics sequencing (Stereo-seq).

**Figure S4. Multiplex spatial imaging and multiplex immunofluorescence landscape.** (A) Familiarity with or use of multiplex spatial imaging platforms. (B) Typical multiplexing capacity in marker-based experiments. (C) Major application areas of multiplex immunofluorescence. (D) Reported challenges in multiplex imaging implementation. Abbreviations: digital spatial profiling (DSP); cyclic immunofluorescence (CyCIF); co-detection by indexing (CODEX); imaging mass cytometry (IMC); multiplexed ion beam imaging (MIBI).

**Figure S5. 3D pathology modality landscape and implementation barriers.** (A) Familiarity with or use of 3D imaging approaches (Question 5.1). (B) Current implementation status of 3D imaging in respondents’ work (Question 5.2). (C) Perceived advantages of 3D imaging over conventional 2D pathology. (D) Reported challenges in 3D pathology implementation.

**Figure S6. Multimodal integration, computational support, future priorities, and clinical translation confidence.** (A) Multimodal integration approaches. (B) Computational support models for spatial and 3D data analysis. (C) Future technological priorities. (D) Expected high-impact biomedical applications. (E) Overall confidence in clinical translation within the next decade (Question 6.5), using all 239 respondents as the denominator and showing no feedback separately. Of 32 no-feedback records, 31 were from non-experts and one from a dual-domain expert; ordered confidence comparisons include 207 valid responses. Abbreviations: artificial intelligence (AI); tumor microenvironment (TME).

**References**

1. Sharma, Akash, Nguyen Tran Minh Duc, Tai Luu Lam Thang, Nguyen Hai Nam, Sze Jia Ng, Kirellos Said Abbas, Nguyen Tien Huy, et al. 2021. “A consensus-based checklist for reporting of survey studies (CROSS).” *Journal of General Internal Medicine* 36:3179−87. <https://doi.org/10.1007/s11606-021-06737-1>

2. Clarsen, Benjamin, Jens Christoffer Skogen, Thomas Sevenius Nilsen, Leif Edvard Aarø. 2021. “Revisiting the continuum of resistance model in the digital age: a comparison of early and delayed respondents to the Norwegian counties public health survey.” *BMC Public Health* 21:730. <https://doi.org/10.1186/s12889-021-10764-2>

3. Newcombe, Robert G. 1998. “Interval estimation for the difference between independent proportions: comparison of eleven methods.” *Statistics in Medicine* 17:873−90. [https://doi.org/10.1002/(SICI)1097-0258(19980430)17:8<873::AID-SIM779>3.0.CO;2-I](https://doi.org/10.1002/(SICI)1097-0258(19980430)17:8%3C873::AID-SIM779%3E3.0.CO;2-I)

4. Benjamini, Yoav, Yosef Hochberg. 1995. “Controlling the false discovery rate: a practical and powerful approach to multiple testing.” *Journal of the Royal Statistical Society: Series B (Methodological)* 57:289−300. <https://doi.org/10.1111/j.2517-6161.1995.tb02031.x>

5. Benjamini, Yoav, Daniel Yekutieli. 2001. “The control of the false discovery rate in multiple testing under dependency.” *The Annals of Statistics* 29:1165−88. <https://doi.org/10.1214/aos/1013699998>

**Full survey questionnaire**

**Current Status of Spatial Biology and 3D Pathology Survey**

**Section 1: Respondent Information**

**1.1 What best describes your primary role?**

Pathologist

Pathology resident

Biomedical engineer

Data scientist/bioinformatician

Industry professional

Clinician (non-pathologist)

Medical school student

Student/Trainee other than medical school

**1.2 How many years of experience do you have in pathology (or primary role above)?**

Less than 2 years

2-5 years

6-10 years

More than 10 years

**1.3   What is your primary work setting?**

Academic medical center

Hospital/clinical laboratory

Biotechnology/pharmaceutical company

Research institute

Government agency

**1.4 Country of practice (where you are currently working/enrolled)**

**1.5 Are you an ASDP (Asian Society of Digital Pathology) member?**

Yes

No

**Section 2: Spatial Biology and 3D pathology Knowledge Assessment**

This section aims to understand your level of awareness and experience, so we can tailor the survey to be more relevant and time-efficient for you.

**2.1.**  **Awareness:** Have you heard of spatial biology and/or 3D pathology?

***Note:*** If you selected "No", you may quit the survey here.
Thank you for your contribution.

Yes

No

**2.2. Experience with Spatial biology:**

On a scale from 1 to 5, what is the extent that you have been working with  spatial biology?
(1: No experience, 5: Expert experience)
**If you select 1,** please proceed directly to **Section 5, question 5.1.**

**2.3. Experience with 3D pathology:**

On a scale from 1 to 5 , what is the extent that you have been working with 3D pathology? 
(1: No experience, 5: Expert experience)

**Section 3: Spatial Transcriptomics**

If you are NOT currently using or NOT familiar with spatial transcriptomics for your research, please proceed directly to **Section 4**

**3.1. Which spatial transcriptomics technologies/brands**  **have you used, currently using, or are familiar with? (Select all that apply)**

10x Genomics Visium

Xenium (in-situ platform)

Nanostring/Bruker GeoMx

CosMx SMI

MERFISH

Slide-seq/Slide-seqV2

Stereo-seq

Spatial ATAC-seq

Other:

**3.2. What is your current level of implementation of spatial transcriptomics in your work?**

Regularly use it in research/clinical work

Have used it occasionally

Working through collaborators

Currently planning to implement it

Interested but haven't implemented yet

**3.3. What do you consider to be the key advantage of spatial transcriptomics over conventional transcriptomics methods?**
(Please choose up to 3 of the following)

Looking for better subtyping (variants) of the disease

Preservation of spatial context and cellular relationships

Ability to study tissue heterogeneity at a finer scale

Identification of novel cell types or states

Correlation with morphological features

Better understanding of disease mechanisms

**3.4. What is the biggest challenge** **you face** **with spatial transcriptomics?**
(Please choose up to 3 of the following)

High cost of technology/reagents

The equipment is unavailable in my region

The equipment is available in my area but not accessible to me

Complex data analysis and integration

Limited spatial resolution

Sample preparation difficulties

Limited gene coverage

Lack of standardized protocols

Lack of expertise/training

**Section 4: Spatial Biology and Multiplex Immunofluorescence (mIF)**

If you are NOT currently using or NOT familiar with spatial biology/mIF, please skip to **Section 5**

(An example of ovarian cancer and immune cells mIF image)

**4.1. Which multiplex imaging technologies/brands** **have you used/currently using or are familiar with?**
(Select all that apply)

Cyclic immunofluorescence (CyCIF)

Multiplexed ion beam imaging (MIBI)

CO-Detection by indEXing (CODEX) /Phenocycler

Imaging Mass Cytometry (IMC)

Digital spatial profiling (DSP)

Vectra Polaris/Phenoptics

MICS imaging

MACSima

COMET

FluoXpert

**4.2. How many markers can you typically multiplex in a single experiment in your work?**

1-5 markers

6-10 markers

11-30 markers

31-50 markers

50+ markers

Not applicable to my work

**4.3. What is your primary application of multiplex immunofluorescence?**
(Please choose up to 3 of the following)

Tumor microenvironment characterization

Immune cell profiling

Biomarker discovery/validation

Spatial mapping of cell-cell interactions

Drug development/therapeutic response prediction

Diagnostic pathology

Clinical trial profiling

**4.4. What is the biggest challenge you face with multiplex immunofluorescence?**
(Please choose  up to 3 of the following.)

Antibody validation and optimization

Signal-to-noise issues

Image acquisition time

Data analysis complexity

Cost of reagents or equipment

Sample preparation issues

Lack of standardization

Limited throughput

The equipment is not available in my region

The equipment is available in my area but not accessible to me

**Section 5: 3D Pathology Research**

 If you are NOT currently using or NOT familiar with 3D pathology, please skip to **Section 6**

**5.1. Which 3D imaging approaches have you used or are familiar with?**
(Select all that apply)

Serial section with 3D reconstruction

Tissue clearing with light sheet microscopy

Tissue clearing with confocal microscopy

Micro-computed tomography (micro-CT)

3D immunohistochemistry/ multiplex imaging (e.g. Akoya Biosciences)

Volumetric pathology imaging platform (z-stack)

Holotomography

Deep learning-based virtual 3D image reconstruction

Volume electron microscopy

Optical projection tomography

**5.2.**  **What is your current level of implementation of 3D imaging in your work?**

Regularly use it in research/clinical work

Have used it occasionally

Currently planning to implement it

Interested but haven't implemented yet

**5.3. What do you consider to be the key advantages**  **of 3D pathology imaging over conventional 2D approaches?**
(Please choose up to 3 of the following)

Better understanding of tissue architecture

Visualization of cell-cell interactions in native context

More accurate quantification of cellular features

Ability to study larger tissue volumes

Enhanced diagnostic accuracy

Nondestructive imaging

Improved clinicopathologic correlation

**5.4. What are the biggest challenges**  **you face with 3D pathology imaging?**  
(Please choose up to 3 of the following)

Equipment cost

Sample preparation complexity

Data storage requirements

Computational demands for image processing

Analysis software limitations

Lack of standardized protocols

Limited throughput

The equipment is not available in my region

The equipment is available in my area but not accessible to me

**Section 6 :** **Multimodal integration, computational data analysis and future directions**

In this section, we invite your expertise on the current integration of spatial biology and 3D pathology with tissue‑level and clinical data (e.g., genomics, imaging, patient records) and its future trajectory, to help identify opportunities and guide next‑generation precision diagnostics and research.

**6.1. Which approaches are you currently using for integrating spatial biology and 3D pathology data with other complementary modalities?** 
(Select all that apply)

Multi-omics integration (e.g., spatial transcriptomics + proteomics, metabolomics, or epigenomics)

Image based integration (e.g., registering H&E WSI with radiology or spatial omics images)

Correlative microscopy (e.g., light microscopy + electron microscopy, confocal with super-resolution imaging)

AI/machine learning for multimodal tissue analysis (tissue imaging and clinical data)

Statistical or computational methods normalization techniques across platforms/modalities

3D tissue reconstruction / volumetric pathology integrated with other modalities

Not currently using multimodal integration

**6.2.  How are you currently handling the computational aspects of spatial/3D data analysis?**
(Please select all applicable)

In-house bioinformatics team

Collaborative partnerships with computational groups

Commercial software solutions

Cloud-based analysis platforms

Open-source software/pipelines

External service providers

Not currently analyzing spatial/3D data

**6.3. In your opinion, which technological advancement would most benefit spatial/3D pathology research?** 
(Please choose up to 3 of the following)

Higher throughput spatial technologies

Lower cost per sample

Improved spatial resolution

Better computational tools for data integration

Standardized analysis workflows

Clinical-grade validation and implementation

Broader marker panels/increased multiplexing capability

AI

Cloud-Based

**6.4. Which application areas in biomedicine do you think will see the most significant impact from spatial/3D technologies in the next 5 years?**
(Please choose up to 5 from the following)

Cancer diagnostics and treatment selection

Enhanced cancer biology insights

Neurodegenerative disease research

Immune-related disorders

Developmental biology

Infectious disease pathology

Digital/computational pathology

Basic tissue biology research

Drug discovery/development

Organoid science

3D Bioprinting

Augmented reality (AR)/virtual reality (VR) imaging for pathology

Patient-specific surgical navigation

Spatial mapping of tumor microenvironments (TME)

**6.5. What is your level of confidence that spatial/3D technologies will become standard in clinical practice, (e.g. in pathology domain, etc.) within the next decade?**
(1: very confident, 5: highly unlikely)

**Section 7: Additional Comments**

Please feel free to add your comments to the following questions. (Optional)

**7.1. What additional technological developments would you like to see in spatial/3D pathological research?**

**7.2.**  **Thank you for taking the time to complete our survey.** Your insights are invaluable and will directly shape how we improve our educational efforts and collaborative projects.  
**Please feel free to provide your feedback about this survey or related topics.**

**7.3.** Optional Acknowledgement:
We would be pleased to recognize your valuable contribution in our presentation at the ASDP 2025 Mumbai Congress. If you wish to be acknowledged, please provide your name and institutional affiliation below. Owing to the substantial number of contributors, we are unable to offer co‑authorship; however, we would be grateful for the opportunity to include your name in our acknowledgements section.
